# Multi-Functional Drug Carrier Micelles With Anti-inflammatory Drug

**DOI:** 10.3389/fchem.2019.00093

**Published:** 2019-02-25

**Authors:** Wei-Jie Wang, Yin-Chou Huang, Chao-Ming Su, Tzong-Rong Ger

**Affiliations:** ^1^Division of Nephrology, Department of Internal Medicine, Taoyuan General Hospital, Ministry of Health and Welfare, Taoyuan, Taiwan; ^2^Department of Biomedical Engineering, Chung Yuan Christian University, Taoyuan, Taiwan

**Keywords:** drug carrier, temperature response micelles, pH response micelles, magnetic micelles, anti-inflammatory drug

## Abstract

The multi-functional micelles poly(*N*-isopropylacrylamide-*co*-*N*,*N*-dimethylacrylamide-*co*-10 undecanoic acid)/CM-Dextran Fe_3_O_4_ (PNDU/CM-Dex Fe_3_O_4_) were poly (NIPAAm-*co*-DMAAm-*co*-UA) (PNDU) grafting hydrophilic CM-Dextran Fe_3_O_4_ which possess pH-dependent temperature response and magnetic response. In this research, anti-inflammation drug Hesperetin was encapsulated by micelles using membrane dialysis method to obtain the different ratio of Hesperetin-embedded P_5_DF_10_, P_10_DF_10_, and P_20_DF_10_. These micelles were characterized by Fourier transform infrared spectroscopy, ^1^H-NMR, thermogravimetric analyzer, and superconducting quantum interference device magnetometer. The morphology and particle size of micelles was observed by transmission electron microscopy and dynamic light scattering. The low critical solution temperature of the P_10_DF_10_ micelles is in pH 6.6 at about 37.76°C and in pH 7.4 at about 41.70°C. The biocompatibility of micelles was confirmed by cytotoxicity study. Inflammatory inhibition of hesperetin-embedded P_10_DF_10_ micelles also studied through RAW264.7. Hesperetin-embed P_10_DF_10_ micelles suppressed LPS-induced inflammatory response. Via immunofluorescence cell staining demonstrate that Hesperetin-embed P_10_DF_10_ micelles inhibited the activation of NF-κB p60 and markedly attenuated in a drug dose-dependent manner. At a concentration of 1,000 ug/ml, an inflammatory rate can be reduced to 36.9%. Based on these results, the hesperetin-embed P_10_DF_10_ micelles had successfully synthesized and enable to carry and release the anti-inflammatory drugs, which instrumental for biomedical therapy and applications.

## Introduction

Inflammation is the common response to injured vascular living tissue. Chemical and physical agents, microbial infections, inappropriate immunological responses or other issues may cause inflammation. The purpose of inflammation is to eliminate the microorganisms, enclose the injury, inactivate toxin so that the tissue or organ can be repaired (Cline M. J., 1970; Morson, 1970). However, some inflammation processes can be an insidious effect on the noxious sensitive reaction in progressive organ damage with the inflammatory mediators especially occur in chronic inflammation (Shin et al., 2008). For example, it is widely accepted that inflammation plays an important role in atherosclerotic plaque formation (Ross, 1999). Some approaches provided to overcome inflammation issue. Pan et al. (2015) presented a useful strategy for the release of anti-inflammatory drug by simply loading enzyme-sensitive polymeric prodrug in the scaffold materials to achieve full-course inhibition of biodegradation-induced inflammatory response. Current approaches mainly focus on the biomodification of the polymeric components for loading of anti-inflammatory drugs (Yoon et al., 2008; Kum et al., 2014; Yuan et al., 2014). Mostly, in order to increase the metabolic activity, inflammation foci will occur in an increase in temperature and an acidic environment (Naghavi et al., 2002). Since inflammation foci could be lead in the integration of some conditions in the living body, it is important in the design of smart responsive anti-inflammatory drug carrier of micelles. It can effectively prevent the disorder of drug delivery as inflammation is being suffered. The smart amphiphilic copolymers are proposed and characterized by their different functionally stimuli-responsive behavior, which is essentially dictated from the functional groups presented by the polymer chain. The most commonly utilized stimuli are temperature, pH, magnetic, light, and ionic interactions. The temperature responsive poly(N-isopropylacrylamide) (PNIPAAm) has lower critical solution temperature (LCST), above LCST the polymer is well dispersed in an aqueous solution or substrate surface, and below LCST the polymer structure deforms for releasing the enclosed drug or surface attached molecules. Guoqing Pan et al. employed an imprinting methodology for PNIPAAm-based molecular of thermo-responsive hydrogel with LCST control for harvesting cell sheets (Pan et al., 2013). PNIPAAm or its copolymers have attracted special attention because of their stable thermal response and employed to solubilize hydrophobic drugs to treatment sites (Soppimath et al., 2005; Schattling et al., 2014). Jia-Shen Wei et al. synthesized poly(N-isopropylacrylamide-co-acrylic acid-co-cholesteryl acrylate) encapsulated anticancer drug for intracellular delivery (Wei et al., 2005). Xiqun Jiang et al. prepared the temperature and pH response micelles of poly (NIPAAm-*co*-acrylic acid)-*b*-poly(caprolactone) to deliver an anti-cancer drug (Zhang et al., 2007). Doo Sung Lee et al. used pH-responsive polymeric micelle based on PEG-poly (β-amino ester)/(amido amine) to detect the cerebral ischemic area (Gao et al., 2011). Subhra Mohapatra et al. and Vronique Prat et al. combined micelles and magnetic particles to deliver a reporter DNA to rat brains (Das et al., 2014) and to derive tumor imaging and therapy (Schleich et al., 2014). Therefore, designing the micelles having a pH-dependent LCST slightly higher than the normal body temperature, drug delivery can be controlled in inflammation foci.

**Figure 1:**
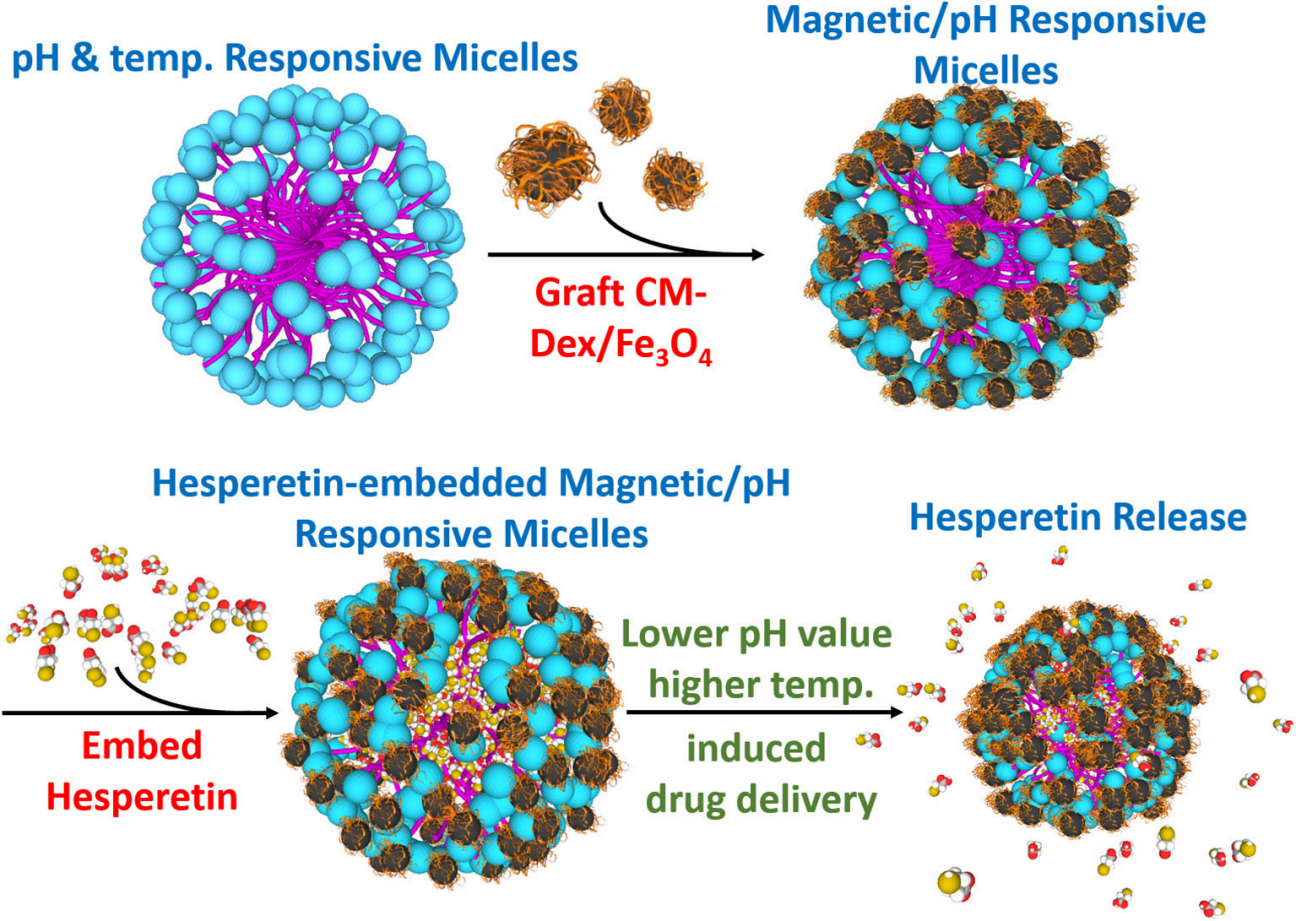
Synthesis diagram of the multi-targeting drug delivery micelle with hesperetin embedded. The multi-targeting micelles are composed of NIPAAm, DMAAm, and UA. The micelles are grafted CM-Dextran/Fe_3_O_4_ for magnetic manipulation. The inflammatory drug hesperetin are embedded into the micelles. Local inflammation area with relatively lower pH value and higher temperature led to the release of drug from the micelles. Target release to the inflammation area could be achieved by external magnetic field.

In this study, the multi-targeting drug carrier micelles were synthesized as shown in Figure 1. The main structure of the multi-targeting drug contained poly *N*-isopropylacrylamide (NIPAAm), *N,N*-dimethylacrylamide (DMAAm), and 10-undecenoic acid (UA). The poly *N*-isopropylacrylamide (NIPAAm), *N,N*-dimethylacrylamide (DMAAm) were synthesized for temperature response, and the 10-undecenoic acids (UA) were synthesized for pH-dependent LCST. The micelles of grafted modified magnetic nanoparticles, CM-Dextran/Fe_3_O_4_, were used as they have magnetic manipulation characteristics for accumulating the micelles in the lesion area. The micelles embed with the anti-inflammatory drug Hesperetin which is a by-product in citrus fruits; as such, it can be easily absorbed by humans (Garg et al., 2001). It is verified to lower cholesterol (Bok et al., 1999; Shin et al., 1999; Kim et al., 2003) and to reduce inflammation (Lin et al., 2005). The treatment strategy for micelles is as which under the normal physiological pH value of 7.4, the LCST is above the normal body temperature of 37°C, leading which to deformation and the release of the enclosed Hesperetin molecules. The lesion area can also be targeted by the external magnetic field through the grafted modified magnetic particle CM-Dextran/Fe_3_O_4_ (CM-Dex/Fe_3_O_4_). Macrophage that has a vascular atherosclerotic inflammatory response was used in this study to verify the drug release test. RAW264.7 macrophages are considered to play essential roles in inflammation which was found to be uniquely effective for measuring inflammatory mediators. This cell line is a good model to study inflammatory responses, which can be activated by lipopolysaccharide (LPS) and trigger the production of inflammatory mediators (i.e., leukotrienes, NF-κB, TNF-α, and ILs) (Rossol et al., 2011; Fan et al., 2013). Among these mediators, the expression of pro-inflammatory cytokine and adhesion molecules is regulated by nuclear factor-κB (NF-κB), which is composed of p50 and p65 subunits (Francisco et al., 2013). To confirm the inflammatory responses in cell staining, we used immunofluorescence method to explore the effects of magnetic micelles on NF-κB activation. As NF-κB is crucially implicated in inflammatory responses, the effects of magnetic micelles on NF-κB activation was explored in activated RAW264.7 macrophages induced by LPS in order to illuminate the anti-inflammatory effect of three magnetic micelles.

## Materials and Methods

### Preparation of Poly (NIPAAm-co-DMAAm-co-UA)/CM-Dex/Fe3O4 Micelles

Firstly, UA was dissolved in an aqueous sodium hydroxide solution (NaOH) and added NIPAAm and DMAAm in solution and stirred uniformly. Hydrochloric acid (HCl) was used to titrate the solution to pH 6.7, and the initiator potassium peroxydisulfate and the chain transfer methyl-3-mercaptopropionate agent were added to conduct a reaction for 4 h. After dialysis and lyophilization, the PNDU-COOCH_3_ was obtained. Since the functional group COOCH_3_ of synthesized PNDU-COOCH_3_ was not easily grafted to magnetic particles, the functional group was modified with NH_2_ by hydrazine. The consecutive step was to modify poly (NIPAAm-*co*-DMAAm-*co*-UA)-COOCH_3_ (PNDU-COOCH_3_) to form poly (NIPAAm-*co*-DMAAm-*co*-UA)-NHNH_2_ (PNDU-NHNH_2_). The PNDU-COOCH_3_, hydrazine and methanol were uniformly stirred for 3 h. After the reaction, dialysis and lyophilization were applied to obtain PNDU-NHNH_2_.

**Figure 2:**
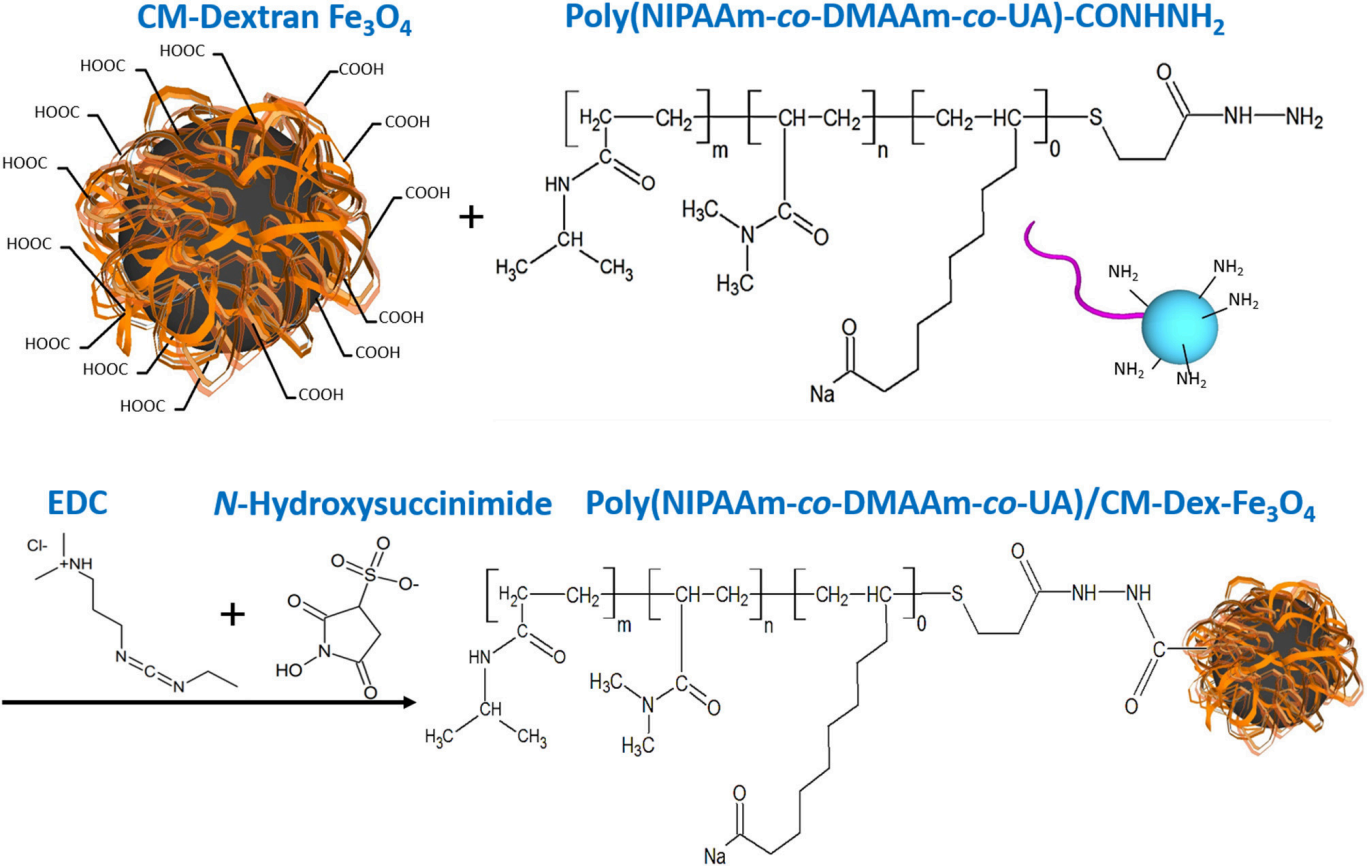
PNDU/CM-Dex-Fe_3_O_4_ chemical equation. First, the carboxyl group on the CM-Dextran/Fe_3_O_4_ was activated, and PNDU-NHNH_2_ was added to enable the carboxyl group, and the amine group to be grafted. EDC and N-Hydroxysuccinimide were used to activate the carboxyl group of CM-Dex/Fe_3_O_4_.

Dextran was a biocompatible, biodegradable and hydrophilic material (Draye et al., 1998), so we synthesize Fe_3_O_4_ with carboxymethyl dextran (CM-Dex). Carboxymethyl dextran magnetic particle (CM-Dex/Fe_3_O_4_) was prepared by mixing ferric chloride (FeCl_3_·6H_2_O), carboxymethyl dextran, hydrazine (N_2_H_4_), and ferrous chloride (FeCl_2_·4H_2_O), and finally obtained by dialysis and lyophilization. When prepared PNDU/CM-Dex-Fe_3_O_4_, the 3-(ethyliminomethyleneamino)-N,N-dimethyl-propan-1-amine (EDC) and N-Hydroxysuccinimide used to activate the carboxyl group of CM-Dex/Fe_3_O_4_, then adds different proportions of PNDU-NHNH_2_ and uniformly stirs it. Further adjusting the molar concentration ratio of PNDU-NHNH_2_ and CM-Dex/Fe_3_O_4_, the purpose of which was to regulate the temperature of the low critical solution temperature (LCST) of the temperature-responsive drug carrier micelles under different pH environments, thereby seeking to meet the temperature of the cardiovascular disease conditions. Then the Poly (NIPAAm-*co*-DMAAm-*co*-UA)/CM-Dex-Fe_3_O_4_ (PNDU/CM-Dex-Fe_3_O_4_) obtained through dialysis and lyophilization. The chemical equation was showed in Figure 2.

### Drug (Hesperetin) Embed Procedure

The drug (Hesperetin) embed procedure was to initially add 30 mg of PNDU/CM-Dex-Fe_3_O_4_ and 15 mg of Hesperetin in *N,N*-dimethylmethanamide (DMF) solution for 2 h, stirred evenly, and then put into 4°C environment for dialysis procedure to be drug-embedded. Collecting the retained solution after dialysis and lyophilization to obtain the micelles embedded Hesperetin.

### Characterization of PNDU/CM-Dex-Fe_3_O_4_ Micelles

The multi-functional drug carrier micelle was characterized by Fourier transform infrared spectroscopy (FT-IR, IR-4200; Jasco, Easton, MD, USA) which was mixed with potassium bromide (KBr), ground into a powder, and made into the pellet. The pellet placed in the FT-IR instrument and scanned wavelength from 400 to 4,000 cm^−1^, the absorption wavelength of the functional group was analyzed. The graft ratio of the micelles would be the quantitative conversion of the integral area measured in Nuclear Magnetic Resonance Spectroscopy (NMR, Ascend 600, Bruker, USA) and the nuclear specie was ^1^H. The morphology of the PNDU/CM-Dex-Fe_3_O_4_ was examined by transmission electron microscope (FEG-TEM, Tecnai F30; Philips, USA). The particle size of the samples was measured by dynamic light scattering (DLS) (LS series, Beckman Coulter, USA).

The critical micelles concentration (CMC) and low critical solution temperature (LCST) were used to evaluate the functionality of micelles. To measure the CMC, the hydrophobic fluorescent agent 1,6-diphenyl-1,3,5-hexatriene (DPH) was embedded in the hydrophobic layer of the PNDU/CM-Dex-Fe_3_O_4_ and measured the fluorescence intensity. The LCST of the multi-functional drug carrier micelles was derived by measuring the permeability of micelles dissolved in Phosphate Buffered Saline (PBS). In order to verify the magnetic properties of the micelles, the micelles were analyzed by the superconducting quantum interference device (SQUID, MPMS 5, Quantum Design, San Diego, CA, USA) and the thermogravimetric analysis (TGA).

### Determination of Drug Loading Efficiency (LE%) and Encapsulation Efficiency (EE%) of Micelles

Dimethylsulfoxide (DMSO) was used to disrupt the micellar structure to be then analyzed spectrophotometrically (ultraviolet-visible [UV-Vis] spectrophotometer; JASCO, Tokyo, Japan) at 231 nm for drug content. The equations we used for the estimation of LE and EE% of Hesperetin in P_5_DF_10_, P_10_DF_10_, P_20_DF_10_ micelles were as follows (Zu et al., 2011; Mohamed et al., 2018):

$$\begin{array}{l} \text{LE}\%=\frac{\text{Hesperetin weight in the micelles}}{Weight\text{ of Hesperetin and polymer added}}\;\times \text{100}\%\\ \text{ EE}\%=\frac{\text{Hesperetin weight in the micelles}}{Weight\text{ of Hesperetin added}}\;\times \text{100}\% \end{array}$$

### Drug (Hesperetin) Release Experiment

The Hesperetin release experiment was to dissolve the lyophilized Hesperetin-embedded PNDU/CM-Dex-Fe_3_O_4_ micelles in a PBS buffer solution of pH 6.6 and pH 7.4, respectively, at a concentration of 1 mg/ml. The two different pH solutions respectively heated to a fixed temperature which were the previously examined LCST. The extracted sample solution and the ethanol solution were configured as test solutions in ethanol/PBS (v:v = 20:80), uniformly stirred, and detected by UV-Vis, and further compared to calculate the release amount of the Hesperetin in micelles.

### Micelles *in vitro* Test

Micelles *in vitro* tests divided into cell viability testing and inflammation assessment. In cell viability testing, PNDU/CM-Dex-Fe_3_O_4_ micelles were co-cultured with L929 mouse fibroblasts and evaluated by MTT-assay. The experimental procedure was first to culture L929 mouse fibroblasts in 24 well-plate, the number of cells was 1 × 10^6^/well. The control group and the experimental group were divided and cultured for 1 day. The plate simultaneously illuminated with UV light to ensure sterility. Then inject culture medium containing PNDU/CM-Dex-Fe_3_O_4_ into the plate of experimental group and co-culture with the cells. After co-culture, the survival co-cultured cells were judged by the absorbance of the control group. The scanning wavelength of 570 nm was set in the ELISA to measure the absorbance of MTT-assay, and the ratio of the experimental group to the control group was the cell viability.

This study used lipopolysaccharide (LPS) to induce the inflammatory response in RAW264.7 mouse macrophages/monocyte and co-culture with multi-functional drug carrier micelles for inflammatory evaluation. The amount of 5 × 10^4^ cells/ml of RAW264.7 mouse macrophage/monocyte was cultured in 96 well-plate, and the positive control group, the negative control group and the experimental group were distinguished. The positive control group contained only macrophage/monocyte and the culture medium; the negative control group was the macrophage/monocyte, the LPS, and the culture medium. The Hesperetin-embed PNDU/CM-Dex-Fe_3_O_4_ micelles were added at 250, 500, and 1,000 μg/ml to the experimental group, respectively, and cultured for 24 h. After the reaction was completed, the scanning wavelength of 540 nm was set in the ELISA to measure the absorbance.

### Immunofluorescence Staining on NF-κB p65

To visualize the expression of NF-κB in cultured RAW264.7, cells were cultured in 24-well plates (1 × 10^6^ cells per well) and pretreated with PNDU/CM-Dex-Fe3O4 micelles P_10_DF_10_ (250, 500, and 1,000 μg/ml) for 1 h at 38°C and stimulated with LPS for 30 min. The cells were then washed and fixed with a 4% paraformaldehyde for 30 min at 37°C, permeabilized with 0.3% Triton X-100 for 15 min, blocked with PBS containing 5% bovine serum albumin (BSA) for 30 min. Next, the cells were processed for immunofluorescent staining with primary NF-κB p65 antibody for 1 h, and followed by incubation with a fluorescein (FITC)-labeled secondary antibody for 1 h before observation. Protein expressed of p65 in RAW264.7 exhibited green fluorescence and observed using a microscope. To create a phase-fluorescence mapping image, the phase contract, and green fluorescence images were overlaid, producing mapping fluorescence in areas of co-localization.

## Results

### Physical and Chemical Properties of Drug Carrier Micelles

This study was based on the pH and temperature response polymer of poly(NIPAAm-*co*-DMAAm-*co*-UA)-COOCH_3_ and it was modified to poly(NIPAAm-*co*-DMAAm-*co*-UA)-NHNH_2_; subsequently. grafting CM-Dextran/Fe_3_O_4_, led to the development of the multi-functional drug carrier micelles of poly(NIPAAM-*co*-DMAAm-*co*-UA)/CM-Dex-Fe_3_O_4_.

**Figure 3:**
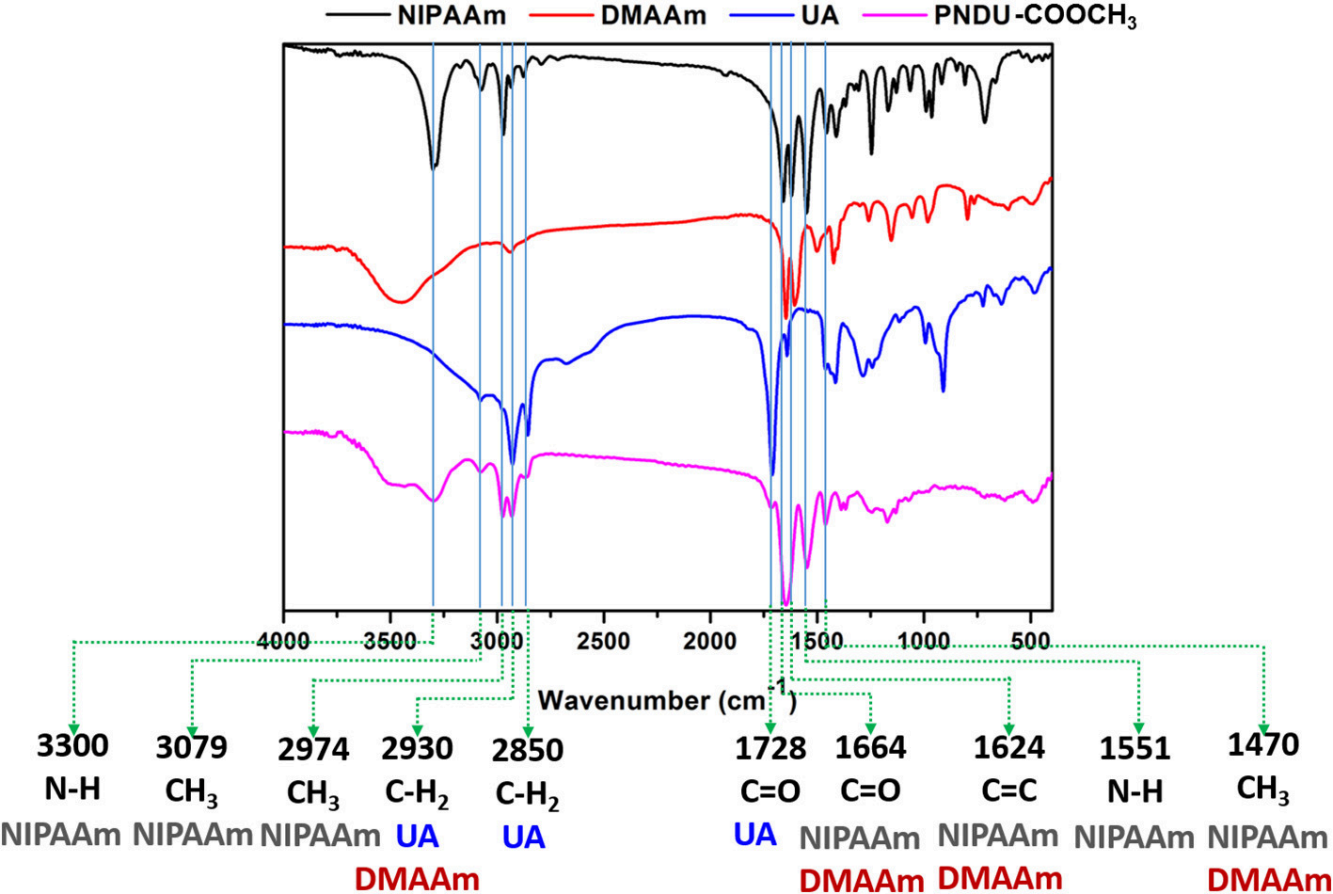
Fourier transform infrared spectroscopy analysis of functional groups of NIPAAm, DMAAm, UA, and PNDU-COOCH_3_. The characteristic peaks of NIPAAm, DMAAm, and UA can be seen in the spectrum of PNDU-COOCH_3_, which proved the successful synthesis.

In this study, FT-IR was used to detect the vibrational absorption peak of the functional group of the material, thereby confirming that the synthesized micelles of PNDU-COOCH_3_. PNDU-COOCH_3_ micelles are synthesized from three monomers: NIPAAm, DMAAm, and UA. Figure 3 shows the FT-IR spectra of NIPAAm, DMAAm, UA, and PNDU. The FT-IR of PNDU were found in the characteristic peaks of NH, CH_3_ and NH of NIPAAm at 3,300, 3,079, 2,974, and 1,551 cm^−1^, respectively. Moreover, the 1,664, 1,728, 2,850, and 2,930 cm^−1^ were the C=O and CH_2_ characteristic peaks of DMAAm and UA, respectively. It was also observed that DMAAm and UA were indeed grafted to PNDU-COOCH_3_. Since both NIPAAm and DMAAm have C=O functional groups, a superposition occurs at 1,664 cm^−1^. At the same time, the C=C functional group of NIPAAm at 1,624 cm^−1^ disappears' this proves that grafting occurs by broken C=C.

**Figure 4:**
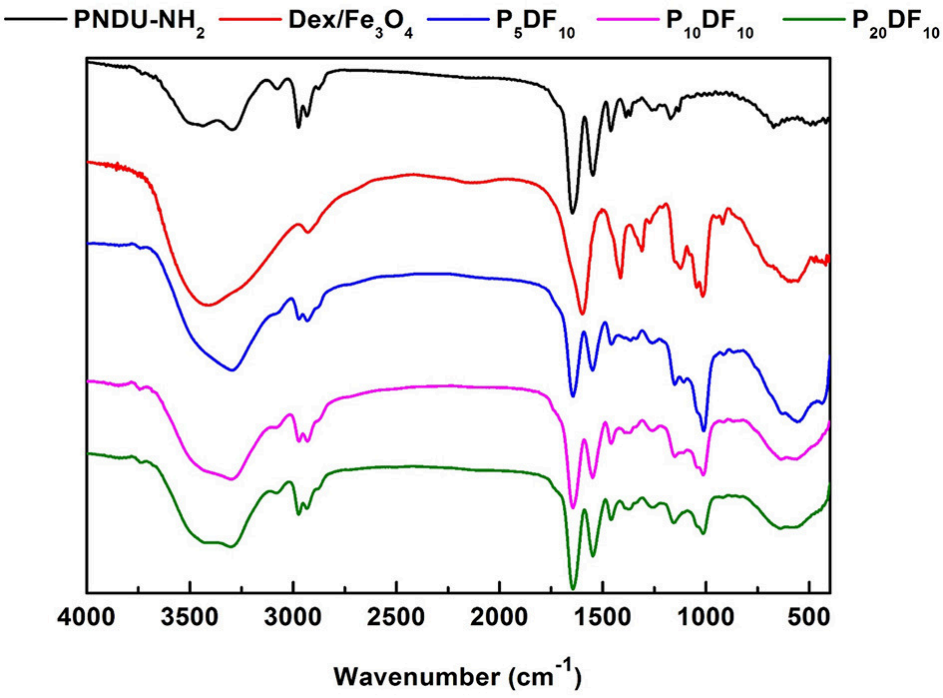
Fourier transform infrared spectroscopy analysis of functional groups of PNDU-NH_2_, CM-Dex/Fe_3_O_4_, P_5_DF_10_, P_10_DF_10_, and P_20_DF_10_. The CM-Dex/Fe_3_O_4_ were successfully grafted onto the PNDU-NH_2_ by Fourier transform infrared spectroscopy.

The PNDU/CM-Dex-Fe_3_O_4_ (PDF) functional group vibration absorption peak was also detected by FT-IR to confirm the synthesized micelles. The PDF micelles were synthesized by PNDU-NH_2_ and CM-Dex/Fe_3_O_4_, and the different ratios were adjusted: PNDU-NH_2_: CM-Dex/Fe_3_O_4_ = 5:10 (P_5_DF_10_), PNDU-NH_2_: CM-Dex/Fe_3_O_4_ = 10:10 (P_10_DF_10_), and PNDU-NH_2_: CM-Dex/Fe_3_O_4_ = 20:10 (P_20_DF_10_). The experimental results are shown in Figure 4; these include spectrum PNDU-NH_2_, Dex/Fe_3_O_4_, P_5_DF_10_, P_10_DF_10_, and P_20_DF_10_.

According to the FT-IR spectrum, 1,728, 1,664, and 1,551 cm^−1^ had the carbonyl group and nitrogen-hydrogen bond functional groups of PNDU-NH_2_, while 2,974, 2,930, and 1,470 cm^−1^ had the carbon-hydrogen bond functional group of PNDU-NH_2_. This indicates that P_5_DF_10_, P_10_DF_10_, and P_20_DF_10_ each have the characteristics of PNDU-NH_2_. The characteristic peaks of the carbon-oxyl and iron-oxyl of CM-Dextran/Fe_3_O_4_ at 1,111, 1,011, and 570 cm^−1^ meant that CM-Dextran/Fe_3_O_4_ had indeed been grafted to PNDU. The higher the iron content of the micelles, the larger the signal. This further indicated that CM-Dextran/Fe3O4 was indeed grafted to the PNDU.

**Figure 5:**
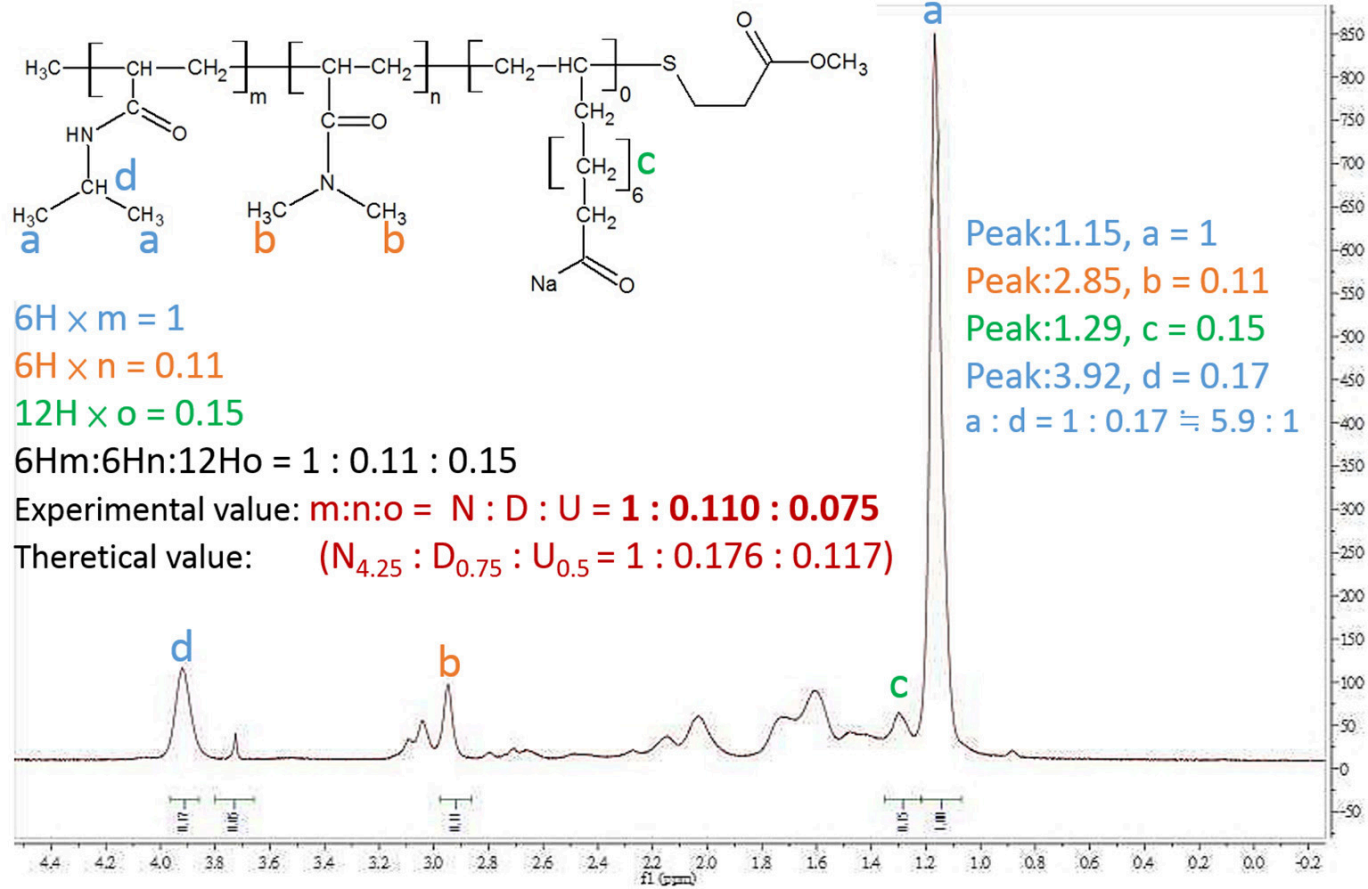
^1^H-NMR spectrum showed the proton signal δ = 1.15 ppm of PNDU-COOCH_3_; δ = 2.85 ppm of NIPAAm, and δ = 1.29 of DMAAm which verified the PNDU-COOCH_3_ composed of NIPAAm, DMAAm, and UA.

The nuclear magnetic resonance spectrometer (^1^H-NMR) was used to confirm the composite of PNDU-COOCH_3_. The ^1^H-NMR spectrum is shown in Figure 5, wherein “a” was the proton signal δ = 1.15 ppm of PNDU-COOCH_3_ and “b” was the proton signal δ = 2.85 ppm of NIPAAm; “c” was the proton signal δ = 1.29 of DMAAm; this verified that the PNDU-COOCH_3_ was polymerized of NIPAAm, DMAAm, and UA.

**Figure 6:**
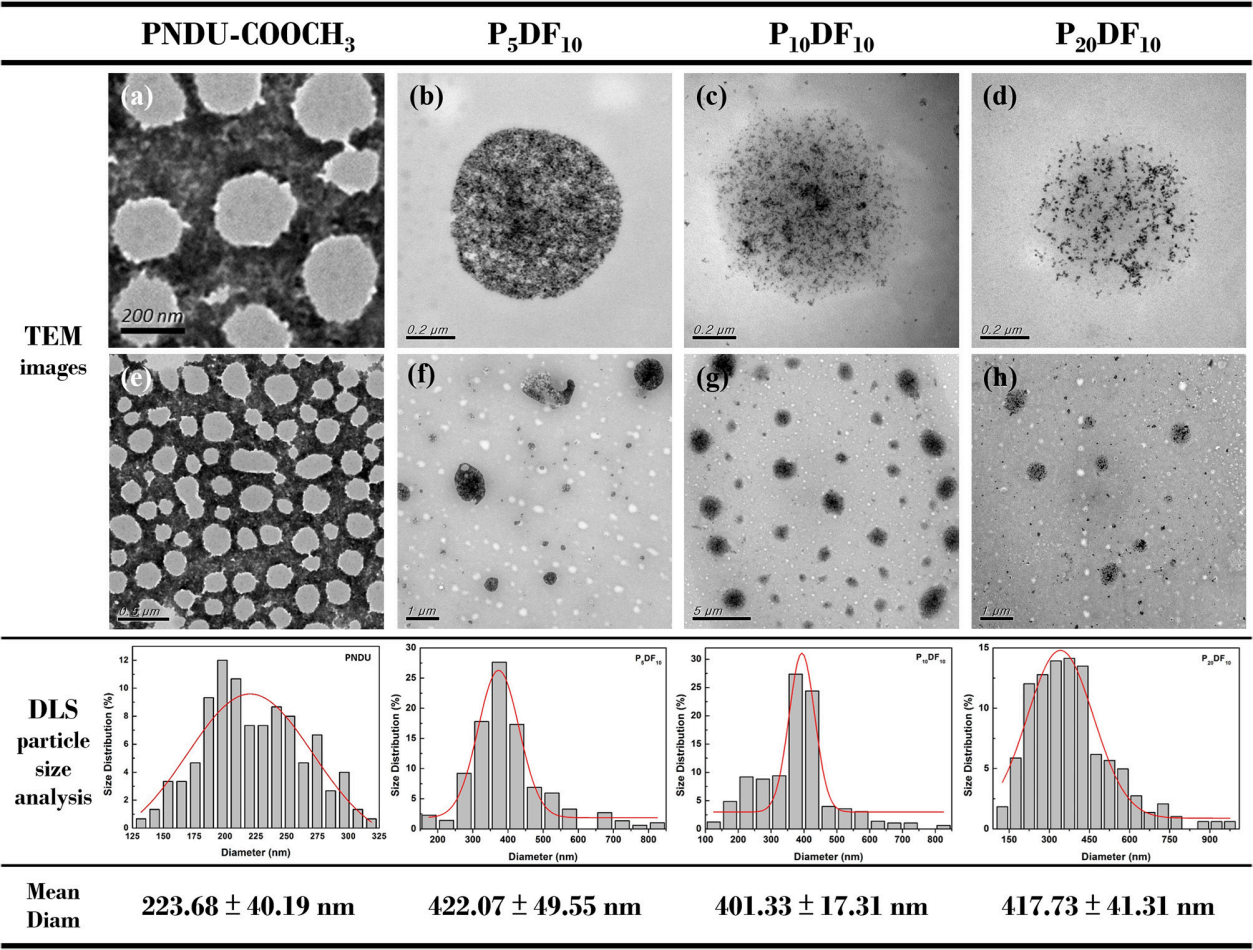
TEM image of **(a,e)** PNDU-COOCH_3_, **(b,f)** P_5_DF_10_, **(c,g)** P_10_DF_10_, and **(d,h)** P_20_DF_10_. The particles size of PNDU, P_5_DF_10_, P_10_DF_10_, and P_20_DF_10_ were 223.68, 422.07, 401.33, and 417.73 nm.

The morphology of PNDU-COOCH_3_, P_5_DF_10_, P_10_DF_10_, and P_20_DF_10_ was examined by TEM and DLS, as shown in Figure 6. The TEM image showed that the higher the iron content of the micelles, the higher the surface density of the iron in the image. The DLS results showed that the average particle size of PNDU-COOCH_3_ was 223.68 ± 40.19 nm; for P_5_DF_10_ it was 422.07 ± 49.55 nm; for P_10_DF_10_ it was 401.33 ± 17.31 nm; and for P_20_DF_10_ it was 417.73 ± 41.31 nm.

### Functional Manifestation of Drug Carrier Micelles

The functional manifestation of the micelles includes the CMC, the LCST and the change in the particle size of the micelles under temperature changes in order to realize the feasibility of the micelles for drug release. Table 1 shows that the LCST of PNDU-COOCH_3_ was pH 6.6: 32.40 ± 1.00°C and pH 7.4: 36.50 ± 0.50°C. This indicated that adding more DMAAm to NIPAAm helped to enhance the effect of the modulating LCST. It was found that the P_10_DF_10_ micelles showed the highest EE% (42.23 ± 2.11) among other samples (as shown in Table 2) and exhibited the LE% of 3.44 ± 0.18. P_5_DF_10_ micelles showed the EE% was 12.55 ± 3.77 and LE% was 2.83 ± 0.21; P_20_DF_10_ micelles showed the EE% was 35.45 ± 5.33 and LE% was 3.51 ± 0.33. Grafting UA allows the amphiphilic polymer to regulate different LCST temperatures due to changes in the pH environment. The LCST of the P_5_DF_10_, P_10_DF_10_, and P_20_DF_10_ LCST were P_5_DF_10_ at pH 6.6: 37.50 ± 1.00°C, pH 7.4: 40.33 ± 1.08°C; P_10_DF_10_ at pH 6.6: 37.76 ± 0.59°C, pH 7.4: 41.70 ± 1.14°C; P_20_DF_10_ at pH 6.6: 35.86 ± 0.51°C, pH 7.4: 39.00 ± 0.52°C. Due to the hydrophilicity of the CM-Dextran/Fe_3_O_4_ on the surface of PNDU-NH_2_, the hydrogen bond on the surface of the micelles became tighter. This made the hydrogen bond less prone to break, it enhanced its hydrophilic properties, and it increased its interaction with water. The LCST of P_5_DF_10_, P_10_DF_10_, and P_20_DF_10_ appeared in higher response temperatures than did PNDU-COOCH_3_.

**Table 1:** The LCST of the PNDU-COOCH_3_, P_5_DF_10_, P_10_DF_10_, and P_20_DF_10_.

**Sample**	**Environmental pH value**	**LCST (°C)**	**ΔT (°C)**
PNDU-COOCH_3_	6.6	32.40 ± 1.00	4.10
	7.4	36.50 ± 0.50	
P_5_DF_10_	6.6	37.50 ± 1.00	2.83
	7.4	40.33 ± 1.08	
P_10_DF_10_	6.6	37.76 ± 0.59	3.94
	7.4	41.70 ± 1.14	
P_20_DF_10_	6.6	35.86 ± 0.51	3.14
	7.4	39.00 ± 0.52	

**Table 2:** Drug loading and encapsulation efficiencies at different ratio of PNDU-Fe_3_O_4_ micelles.

**Sample**	**LE%**	**EE%**
P_5_DF_10_	2.83 ± 0.21	12.55 ± 3.77
P_10_DF_10_	3.44 ± 0.18	42.23 ± 2.11
P_20_DF_10_	3.51 ± 0.33	35.45 ± 5.33

### PDF Micelles Magnetic Verification and Magnetic Content Analysis

**Figure 7:**
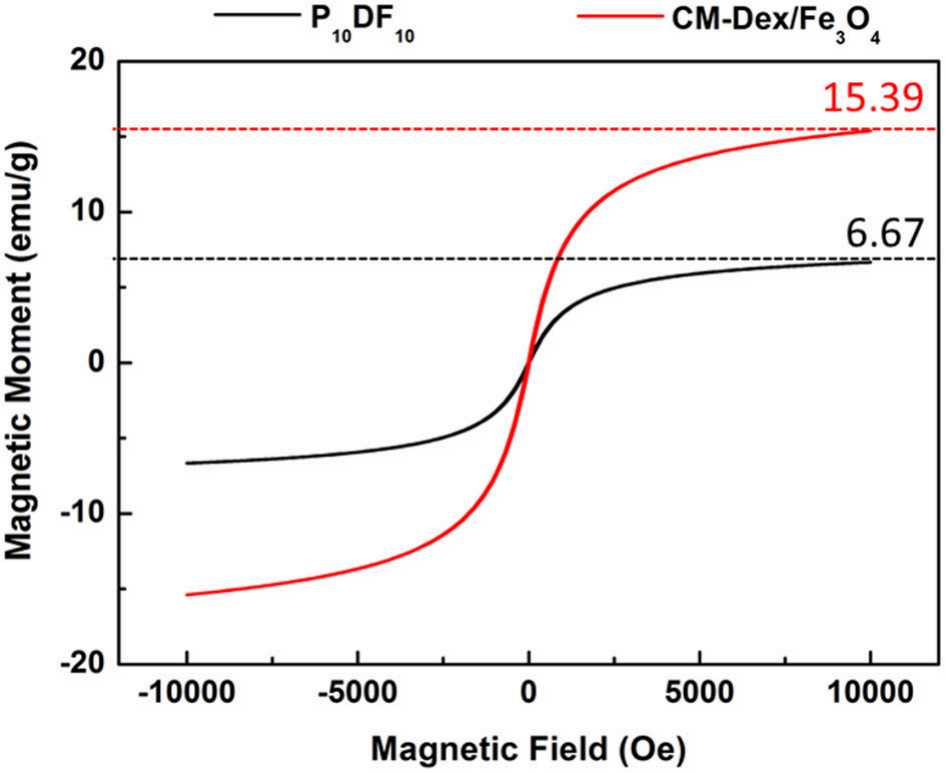
SQUID verification of CM-Dex/Fe_3_O_4_ and P_10_DF_10_. The saturation magnetic moment of CM-Dex/Fe_3_O_4_ and P_10_DF_10_ were 15.39 emu/g and 6.67 emg/g which represented the P_10_DF_10_ contained 43.33% CM-Dex/Fe_3_O_4_.

This study verified that PDF micelles have magnetic properties for magnetic targeting. The saturation magnetization results detected by SQUID are shown in Figure 7. The magnetic moment of CM-Dex/Fe_3_O_4_ is 15.39 emu/g, while the magnetic moment of the P_10_DF_10_ micelles is 6.67 emu/g; this verified that magnetic property was contained. The content of CM-Dex/Fe_3_O_4_ in P_10_DF_10_ could be estimated by the ratio of the saturation magnetic moment of the CM-Dex/ Fe_3_O_4_ and P_10_DF_10_ micelles. The ratio between CM-Dex/Fe_3_O_4_ and P_10_DF_10_ was 43.33%, which indicated that the P_10_DF_10_ contained 43.33% CM-Dex/Fe_3_O_4_. This result was greatly consistent with the proportion of P_10_DF_10_ (PNDU-NH_2:_ CM-Dex/Fe_3_O_4_ = 10:10).

**Figure 8:**
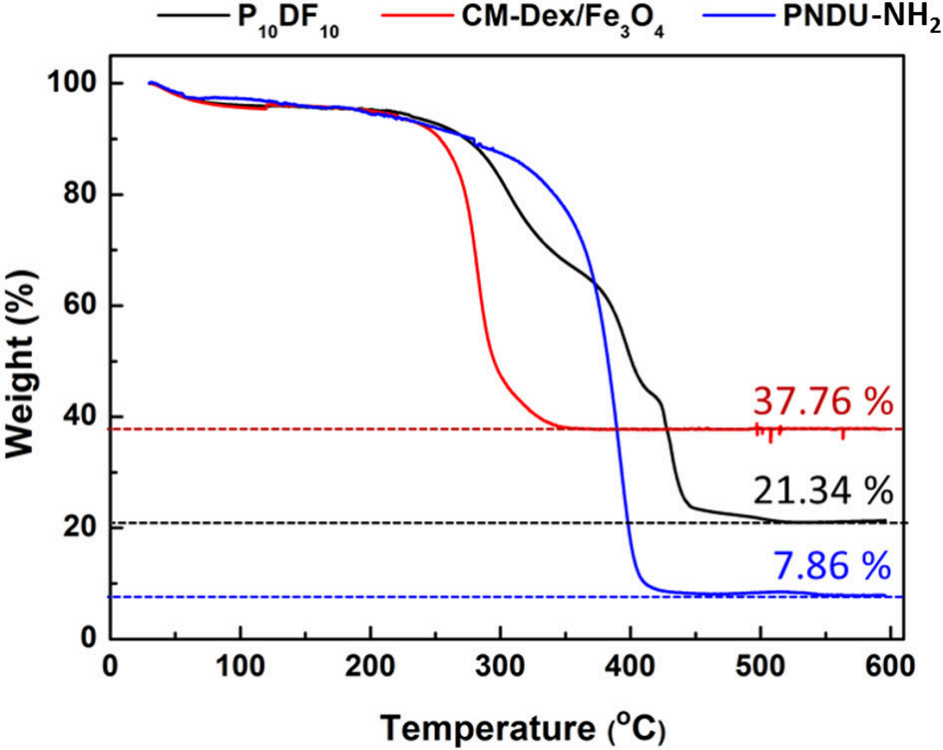
P_10_DF_10_ TGA analysis. The residual amount of CM-Dextran/Fe_3_O_4_, P_10_DF_10_, and PNDU-NH_2_ was 37.76, 21.34, and 7.86% that extrapolated the content of CM-Dextran/Fe_3_O_4_ in P_10_DF_10_ was 45.08%.

Thermo-gravimetric analysis was used to predict the structure and composition of the material. In this study, TGA was used to investigate the synthesis ratio of grafted CM-Dex/Fe_3_O_4_. The TGA results, shown in Figure 8, reflect that the residual amount of CM-Dextran/Fe_3_O_4_, P_10_DF_10_, and PNDU-NH_2_ was 37.76, 21.34, and 7.86%. The P_10_DF_10_ was composed of PNDU-NH_2_ and CM-Dextran/Fe_3_O_4_, and that each proportion of the composition could be extrapolated from the residual amount. The results of the content of CM-Dextran/Fe_3_O_4_ in P_10_DF_10_ were 45.08%, which was similar to those found in the SQUID analysis.

### Drug (Hesperetin) Release Experiment

**Figure 9:**
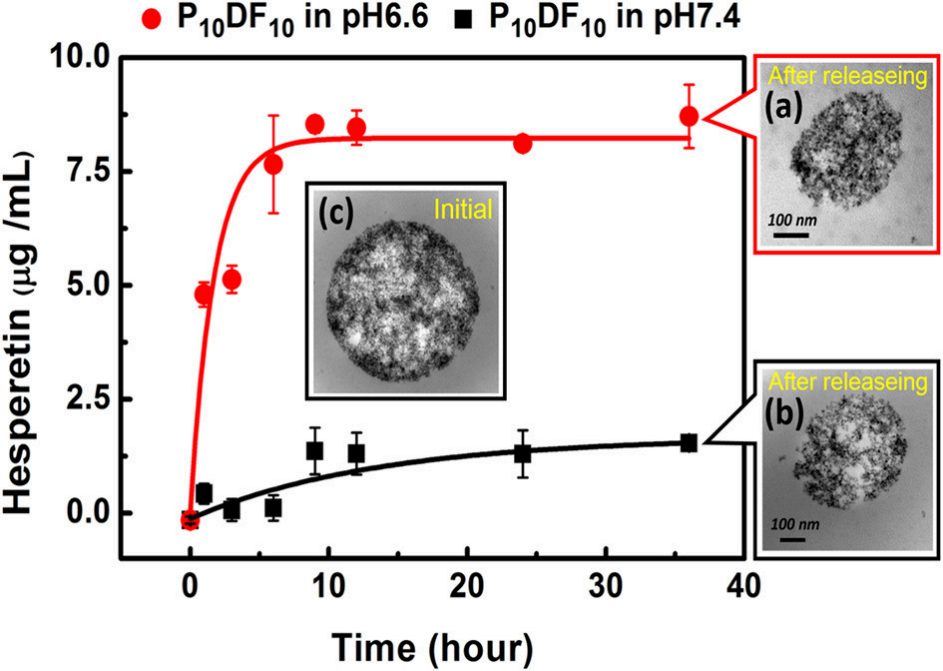
Drug release experiment of hesperetin-embed P_10_DF_10_ micelles. **(a)** was the TEM image of the hesperetin-embed P_10_DF_10_ micelles released drug at pH 6.6 at 39°C; **(b)** was TEM image of the hesperetin-embed P_10_DF_10_ micelles at pH 7.4 at 39°C; **(c)** was the initial TEM image of hesperetin-embed P_10_DF_10_ micelles.

The drug carrier Hesperetin-embed P_10_DF_10_ micelles controlled the release rate of the drug at different pH values through its LCST characteristics. The purpose was to release the drug (Hesperetin) at the inflammatory area wherein the temperature is slightly above that of the normal tissue; thus, if it was at the same temperature as the normal tissue, the drug was protected inside the micelles. The micelles drug release amounts at 1, 3, 6, 9, 12, 24, and 36 h of pH 6.6 at 39°C were 4.79816, 5.13189, 6.28425, 7.66239, 7.6419, 7.93533, and 8.59221 μg. The micelles drug release amounts at 1, 3, 6, 9, 12, 24, and 36 h of pH 7.4 at 39°C were 0.42561, 0.06561, 0.11299, 1.36382, 1.306, 1.29812, and 1.53725 μg, as shown in Figure 9.

It can be seen from the results that when the ambient temperature was higher than the LCST, the drug (Hesperetin) was released due to the change of the micelles morphology. When the release time was ~9 h, the cumulative release amount reached a saturated state.

### Micelles *in vitro* Test

**Figure 10:**
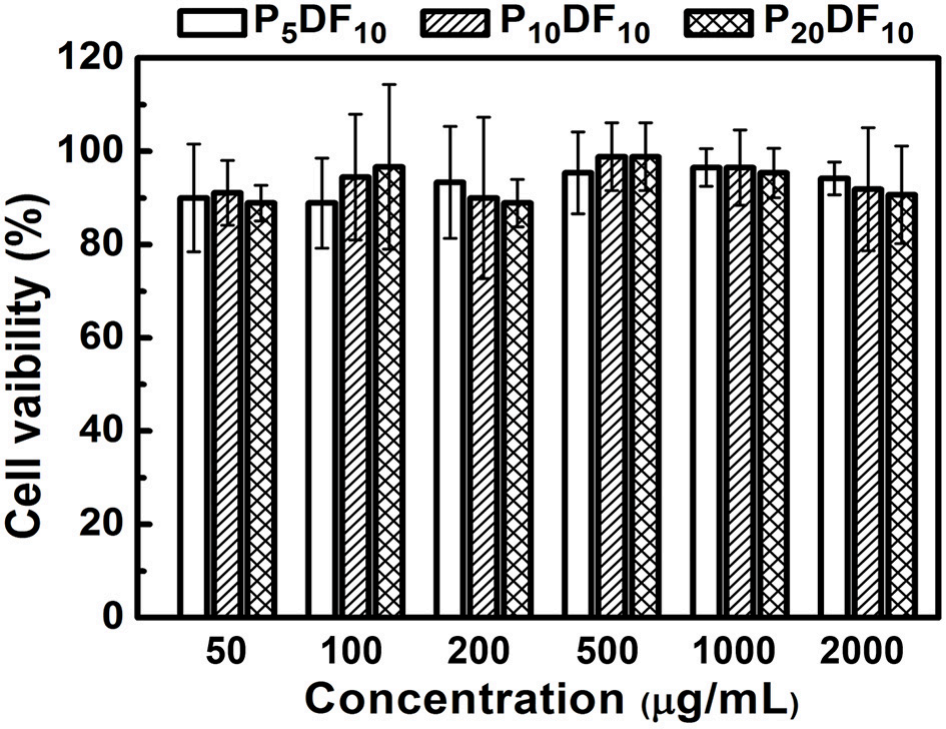
The cell viability test of P_5_DF_10_, P_10_DF_10_, and P_20_DF_10_. The cell viability was around 90% in different intake concentration of P_5_DF_10_, P_10_DF_10_, and P_20_DF_10_ that showed the good biocompatibility of micelles.

The micelles *in vitro* test was divided into two parts: the cell viability test and the inflammatory effect. A L929 mouse fibroblast was used for the cell viability test, and a RAW264.7 mouse macrophage/monocyte was used for the inflammatory response. As shown in Figure 10, the cell viability was ~90%. For an intake concentration of 50 μg /mL, the cell viability of P_5_DF_10_, P_10_DF_10_, and P_20_DF_10_ were 90, 91.11, and 88.88%; in a drug intake concentration 100 μg /mL, the cell viability of P_5_DF_10_, P_10_DF_10_, and P_20_DF_10_ were 88.89, 94.44, and 96.66%; in a drug intake concentration 200 μg/mL, the cell viability of P_5_DF_10_, P_10_DF_10_, and P_20_DF_10_ were 93.33, 90, and 88.88%. These results showed that P_5_DF_10_, P_10_DF_10_, and P_20_DF_10_ all had good biocompatibility and low toxicity to L929 mouse fibroblasts.

**Figure 11:**
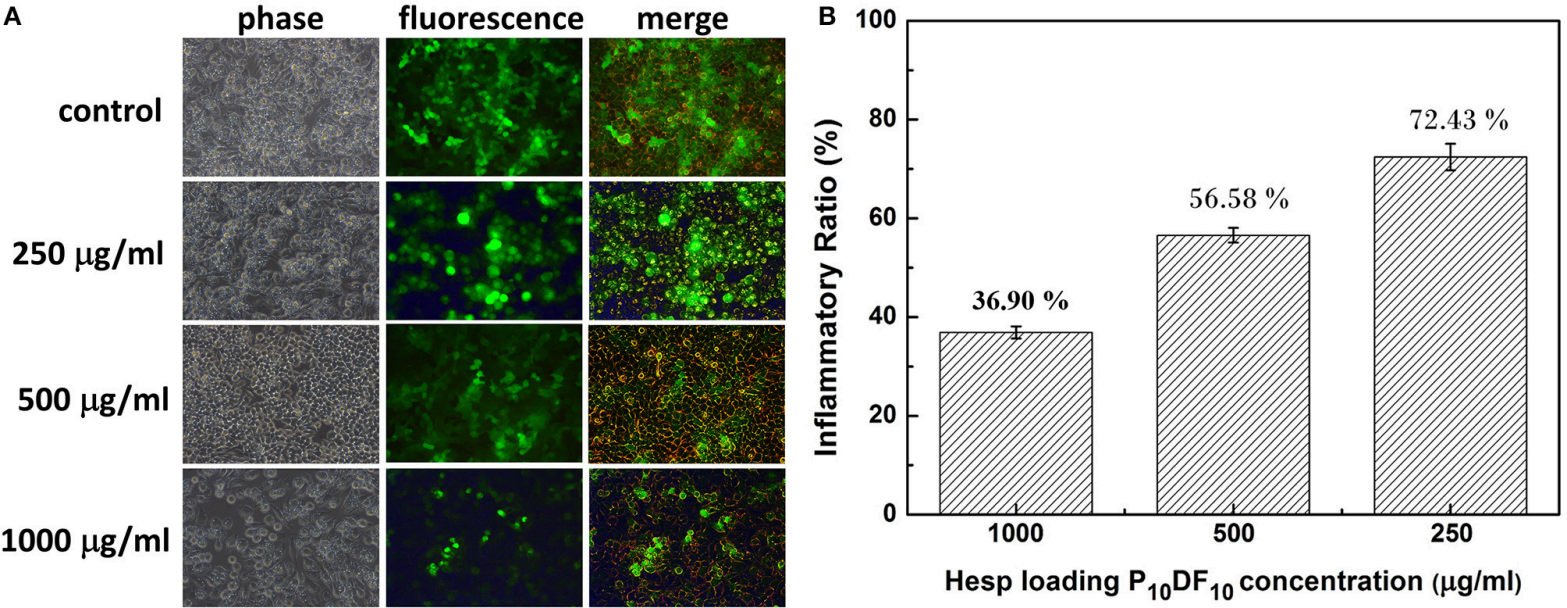
**(A)** P_10_DF_10_ prevented LPS-induced translocation of NF-κB p65 subunit by immunofluorescence studies. Cells were treated without P_10_DF_10_ (control) or with P_10_DF_10_ of 250, 500, and 1,000 μg/ml for 1 h and stimulated with LPS for 30 min, fixed, and incubated with anti-p65 antibody followed by FITC conjugated second antibody (green fluorescence). **(B)** Anti-Inflammatory experiment of the RAW264.7. The inflammatory ratio in the Hesperetin-embed P_10_DF_10_ micelles concentration at levels of 1,000, 500, and 250 μg/mL were 36.90, 56.58, and 72.43%. Data indicated that higher concentrations of Hesperetin-embed P_10_DF_10_ micelles could get higher anti-inflammatory ability.

In addition to their drug release function, the multi-functional drug carrier micelles are expected to reduce inflammation in cell tissues. Figure 11 showed the Hesperetin-embed P_10_DF_10_ micelles in cell study to confirm the anti-inflammatory property. By immunofluorescent staining of p65, we observed that p65 was exclusively distributed in the cytoplasmic and nucleus after LPS stimulation, as shown in Figure 11A. The nuclear translocation of p65 was markedly attenuated in a dose-dependent manner by Hesperetin-embed P_10_DF_10_ micelles treatment from 250, 500, to 1,000 μg/ml. These results indicated the potential role of NF-κB in the suppression of inflammatory mediators-TNF-α and IL-6 production by Hesperetin-embed P_10_DF_10_ micelles. Figure 11B showed the Hesperetin-embed P_10_DF_10_ micelles and the RAW264.7 were used in the inflammatory response test. RAW264.7 cells show non-inflamed to negative control, while an inflammatory ratio 100% to positive controls. The inflammatory ratio in the Hesperetin-embed P_10_DF_10_ micelles concentration at levels of 1,000, 500, and 250 μg /mL were 36.90, 56.58, and 72.43%. The results showed that the drug Hesperetin was released into the inflammatory cells and that the higher the concentration of Hesperetin-embed P_10_DF_10_ micelles was, the more it inhibited inflammation.

## Discussion

The multi-functional drug carrier micelles were synthesized by PNDU-NH_2_ and CM-Dextran/Fe_3_O_4_, and the functional groups were confirmed by FT-IR and ^1^H-NMR. TGA and SQUID verified the CM-Dextran/Fe_3_O_4_ ratio and the magnetic properties of the micelles and we observed the morphology of micelles by using TEM. This study regulated the composition of CM-Dextran/Fe_3_O_4_ and PNDU-NH_2_ to optimize LCST finding that it was suitable for the lesion area at a slightly higher temperature than normal tissues; it also responded to the environments of normal tissue (pH7.4) and lesions (pH6.6), which confirmed that the micelles had pH and temperature response behaviors. The drug Hesperetin was selected in this study as the drug carrier to reduce the oxidation of LDL with the aim of reducing the occurrence of atherosclerosis; in addition, it was expected to increase the therapeutic effect as well. The Hesperetin released from the micelles reached saturation after 9 h, and the release amount of pH6.6 and pH7.4 after 36 h were 8.59221 μg and 1.53725 μg, respectively. This achieved the aim of drug release in the lesion area. The *in vitro* test for cell viability and inflammation assessment was investigated. A L929 mouse fibroblast and P_5_DF_10_, P_10_DF_10_, and P_20_DF_10_ were used in a cell viability test. The cell viability was around 90% in concentrations of 200, 100, and 50 μg/ml and it possessed good biocompatibility. The RAW264.7 macrophage/monocyte was used in the inflammatory response test. At a concentration of 1,000 μg/ml, the inflammatory ratio can be effectively reduced to 36.9%, which realizes the function of the drug carrier micelles. The main challenging task is to develop a drug deliver micelles capable of responding to such a narrow range of pH-dependent LCST in inflammation foci. The micelles synthesized in this study showed the good outcomes form the LCST results and anti-inflammation drug releasing tests in a narrow window of pH change of 6.6 and 7.4, which compared other micelles pH-dependent LCST responding to different pH value of 5.5 and 7.4 (Yu et al., 2014) and pH value of 5 and 7.4 (Chen et al., 2013). In addition, magnetic nanoparticle grafted on PNDU micelles provided a higher magnetic property which was 6.67 emu/g, than others MNPs-loaded micelles 0.06 emu/g (Yang et al., 2016), 1.71 emu/g (Naous et al., 2017) it promoted the potential of magnetic targeting or other manipulating application.

## Conclusion

This study successfully provided multi-functional drug carrier Hesperetin-embed micelles with good biocompatibility, while maintaining the characteristics of PH-dependent temperature response, and magnetic properties. The drug carrier magnetic micelles were synthesized by PNDU-NH_2_ and CM-Dextran/Fe_3_O_4_, and the functional groups were confirmed. This study regulated the composition of CM-Dextran/Fe_3_O_4_ and PNDU-NH_2_ to optimize LCST finding in P_10_DF_10_ that it was suitable for the lesion area at a slightly higher temperature than normal tissues. In the drug hesperetin release experiment, P_10_DF_10_ micelles approximately higher 5.7 times in pH6.6 than in pH7.4 showed that using these magnetic micelles, moderately hydrophobic drugs can selectively deliver to acidic inflammation foci. Its iron content was nearly about 45% and it was provided magnetic properties (6.67 emu/g), it promoted the potential of magnetic targeting. Data provided the evidence for suggesting that Hesperetin-embed P_10_DF_10_ micelles suppressed LPS-induced inflammatory response. Via immunofluorescence cell staining demonstrate that Hesperetin-embed P_10_DF_10_ micelles inhibited the activation of NF-κB p60 and markedly attenuated in a drug dose-dependent manner. At a concentration of 1,000 μg/ml in Hesperetin-embed P_10_DF_10_ micelles, the inflammatory ratio can be effectively reduced to 36.9%, suggesting that these magnetic micelles can be a good candidate for anti-inflammatory therapy in the further clinical trial.

## Author Contributions

W-JW contributed substantially to the conception and selection of the drug in micelles. W-JW is the PI of the project of magnetic micelles application with hesperetin treatment from Taoyuan General Hospital, Ministry of Health and Welfare. W-JW also assisted in carrying out *in vitro* tests with a magnetic micelles dosage for inflammatory study. Y-CH is the master student from Prof. Ger's lab and has significant experience in synthesizing temperature-responsive magnetic micelles and shares first authorship. C-MS played an important role in PNDU synthesis and was of great support for the development of pH responsive micelles. T-RG is the research adviser of Y-CH and C-MS, and is also a co-PI of the project from Taoyuan General Hospital, Ministry of Health and Welfare. He also wrote this manuscript.

### Conflict of Interest Statement

The authors declare that the research was conducted in the absence of any commercial or financial relationships that could be construed as a potential conflict of interest.
